# The In Vitro Effects of Enzymatic Digested Gliadin on the Functionality of the Autophagy Process

**DOI:** 10.3390/ijms19020635

**Published:** 2018-02-23

**Authors:** Federico Manai, Alberto Azzalin, Fabio Gabriele, Carolina Martinelli, Martina Morandi, Marco Biggiogera, Mauro Bozzola, Sergio Comincini

**Affiliations:** 1 Department of Biology and Biotechnology, University of Pavia, 27100 Pavia, Italy; federico.manai01@universitadipavia.it (F.M.); azzalin.alberto@gmail.com (A.A.); fabio.gabriele01@universitadipavia.it (F.G,); carolina.martinelli991@gmail.com (C.M.); martina.morandi01@universitadipavia.it (M.M.); marco.biggiogera@unipv.it (M.B.); 2 Pediatrics and Adolescentology Unit, Department of Internal Medicine and Therapeutics, University of Pavia, Fondazione IRCCS San Matteo, 27100 Pavia, Italy; mauro.bozzola@unipv.it

**Keywords:** celiac disease, gluten, autophagosome, Caco-2 cells

## Abstract

Gliadin, the alcohol-soluble protein fraction of wheat, contains the factor toxic for celiac disease (CD), and its toxicity is not reduced by digestion with gastro-pancreatic enzymes. Importantly, it is proved that an innate immunity to gliadin plays a key role in the development of CD. The immune response induces epithelial stress and reprograms intraepithelial lymphocytes into natural killer (NK)-like cells, leading to enterocyte apoptosis and an increase in epithelium permeability. In this contribution, we have reported that in Caco-2 cells the administration of enzymatically digested gliadin (PT-gliadin) reduced significantly the expression of the autophagy-related marker LC3-II. Furthermore, electron and fluorescent microscope analysis suggested a compromised functionality of the autophagosome apparatus. The rescue of the dysregulated autophagy process, along with a reduction of PT-gliadin toxicity, was obtained with a starvation induction protocol and by 3-methyladenine administration, while rapamycin, a well-known autophagy inducer, did not produce a significant improvement in the clearance of extra- and intra-cellular fluorescent PT-gliadin amount. Altogether, our results highlighted the possible contribution of the autophagy process in the degradation and in the reduction of extra-cellular release of gliadin peptides and suggest novel molecular targets to counteract gliadin-induced toxicity in CD.

## 1. Introduction

The term “autophagy”, originally coined by De Duve [1], is a self-degradative process crucial for eukaryotic cells for balancing sources of energy during development phases and in response to different environmental conditions [2]. Autophagy also plays a cell-protective function in removing misfolded or aggregated proteins, clearing damaged organelles [3], as well as eliminating intracellular pathogens [4]. In general, autophagy can be considered a survival mechanism, with the possibility, under certain circumstances, to induce a programmed cell death fate [5]. Autophagy can be divided into a non-selective or selective process for the catabolic degradation of organelles, ribosomes and protein aggregates [6]. Autophagy plays also a key role to control inflammation through the inhibition of the inflammasome, antigen presentation, T cell homeostasis, and the secretion of immune mediators [7]. Autophagy impairment is crucial in several diseases, in particular proteopathies, such as Alzheimer [8], Parkinson [9], and Huntington diseases [10].

Celiac disease (CD) is a chronic systemic autoimmune disease of the small intestine with genetic and environmental components. This disease originates from a pathological immune response to ingested dietary food-derived digested gluten from different cereals like wheat, barley, and rye [11]. In particular, the causative agent in wheat is the ethanol soluble prolamin fraction, i.e., gliadin, whose toxicity remains after peptic-tryptic (PT) digestion [12]. Gluten contains hundreds of prolamin proteins, in the form of mono-, oligo-, or polymers linked by interchain disulphide bonds. Furthermore, gliadins can be subdivided into four different types, according to their aminoacids composition and to their molecular weights, specifically ω5-, ω1,2-, α/β-, and γ-gliadins [13]. Several gliadin epitopes show different immunogenic and toxic properties. These epitopes present multiple proline and glutamine residues that give rise to resistance to proteolysis by gastric, pancreatic, and intestinal proteases. Importantly, glutamine residues are targets for deamination by tissue transglutaminase (TG2), leading to an increased immunogenicity [14]. Currently, the only therapeutic option for CD patients is a defined and restricted a gluten-free diet (GFD). However, following a GFD is cumbersome [15], as a recent study from the UK indicates that the large majority of diagnosed CD patients introduced gluten either intentionally or inadvertently in their diets [16]. Furthermore, in other CD patients, GFD was not completely effective to suppress the disease symptoms [17].

In this contribution, the effect of enzymatic digested gliadin molecules on the functionality of the autophagy process was investigated in Caco-2 cells, a widely adopted model system that resemble intestinal epithelial permeability in CD studies.

## 2. Results

### 2.1. PT-Gliadin Aggregation and Internalization in Caco-2 Cells

The results were focused on the evaluation and modulation of the autophagy process, in order to counteract gliadin cytotoxicity in Caco-2 cells.

**Figure 1:**
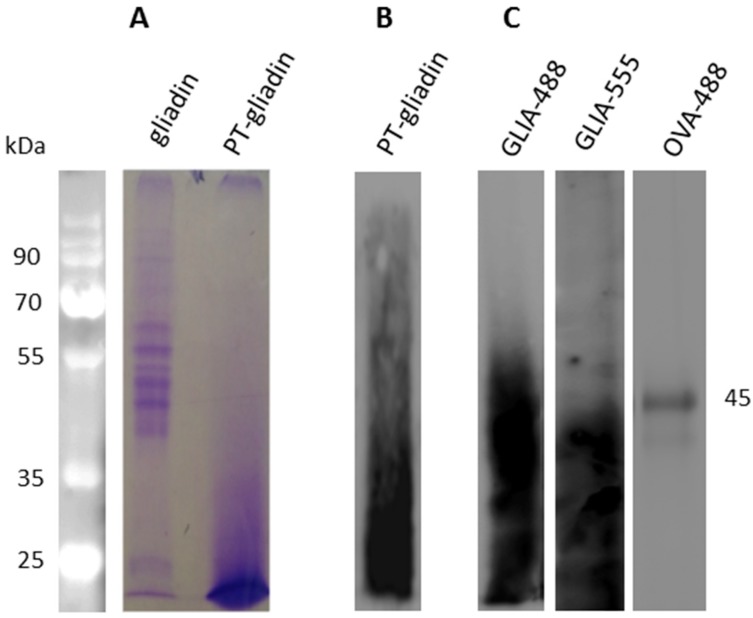
Electrophoretic characterization of enzymatically digested (PT)-gliadin fragments after peptic-tryptic digestion. (**A**) Protein gel electrophoresis after Coomassie staining of whole gliadin and PT-gliadin (both 100 µg) (**B**) Immunoblotting analysis of PT-gliadin (40 µg) using a mouse monoclonal anti-gliadin antibody; and, (**C**) Protein gel electrophoretic analysis of fluorescently labelled PT-gliadin (GLIA-488 and GLIA-555) and ovalbumin (OVA-488) proteins (40 µg). MW, in kDa are reported.

Firstly, commercial wheat gliadin was subjected to an enzymatic peptic-tryptic digestion to mimic the normal intestinal degradation according to previous work [18]. The obtained digested protein products (i.e., PT-gliadin) were visualized by protein gel electrophoresis (Figure 1A) and immunoblotting (Figure 1B) showing for the digested gliadin proteins (PT-gliadin) a hetero-dispersed pattern ranging from few to 40 kDa. Next, PT-gliadin and egg ovalbumin proteins were covalently labelled with the fluorescent dyes Alexa Fluor 488 or Alexa Fluor 555 (thus producing GLIA-488, GLIA-555 and OVA-488, respectively) (Figure 1C), in order to better monitor and quantify the uptake and the release of PT-gliadin and ovalbumin proteins in Caco-2 proliferating cells.

**Figure 2:**
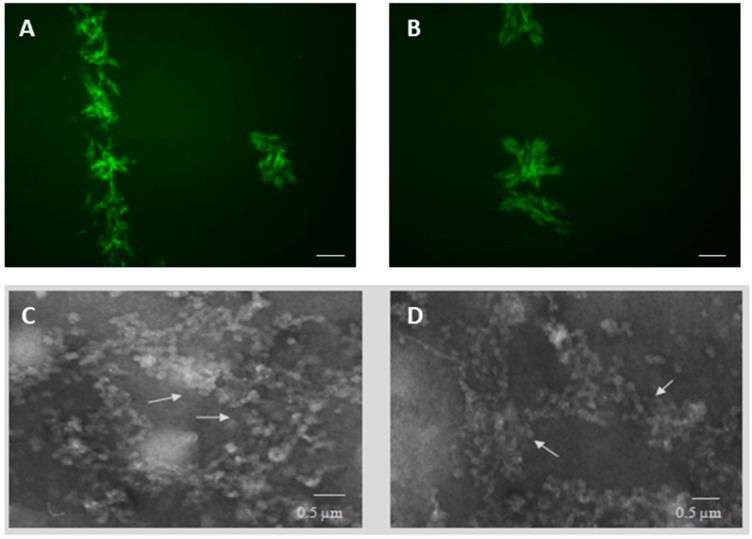
PT-gliadin fluorescent and ultrastructural visualization. (**A**,**B**) Labelled PT-gliadin (GLIA-488, 10 µg) was added in 1 mL DMEM medium and visualized by fluorescent inverted microscope Eclipse Nikon TS100 (scale bars = 10 µm). (**C**,**D**) TEM evaluation of PT-gliadin aggregates, indicated by arrows.

Subsequently, labelled or unlabelled PT-gliadin proteins were visualized using fluorescent and electronic microscope (TEM), respectively. As highlighted by inverted fluorescent microscope analysis (Figure 2A,B), PT-gliadin (GLIA-488) was able to form spontaneous and relatively complex aggregates in cell-culture medium, as already reported [19]; differently, OVA-488 was barely visible and did not produced evident aggregates (data not shown). Similarly to fluorescent evidences, intra-molecular aggregates of PT-gliadin were documented by TEM analysis (Figure 2C,D).

**Figure 3:**
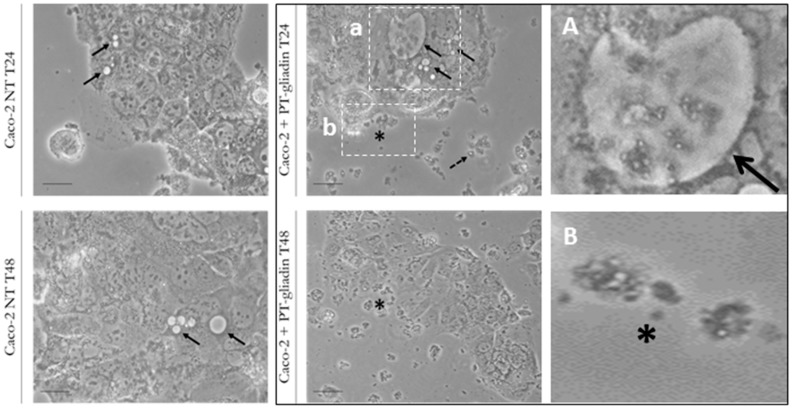
Morphological evaluation of PT-gliadin administration in Caco-2 cells by optical microscopy analysis. Caco-2 cells were incubated with PT-gliadin (**right**, 1 µg/µL) or not- (NT) (**left**) and visualized using inverted optical microscope Eclipse Nikon TS100 at 24–48 h p.t. Continuous arrows indicate intra-cellular vesicles, dashed arrows point for extra-cellular PT-gliadin aggregates in proximity to the plasma membranes (asterisks) (scale bars = 10 µm). (**A**,**B**) are magnifications of **a** and **b** panels, respectively.

**Figure 4:**
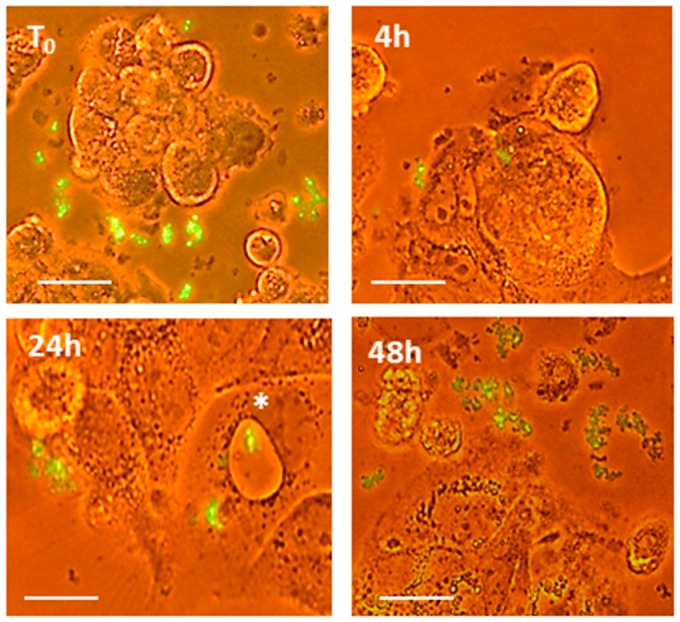
Internalization and extra-cellular release of fluorescent PT-gliadin in Caco-2 cells. Analysis of GLIA-488 traffic in Caco-2 cells through fluorescent inverted microscope Eclipse Nikon TS100 at T_0_–48 h p.t. Visualizations were performed through an inverted microscope Eclipse Nikon TS100, 40× objective. Asterisk indicates a large intra-cellular vesicle containing large GLIA-488 aggregates (scale bars = 30 µm).

To investigate the morphological and functional effects induced by PT-gliadin uptake, proliferating Caco-2 cells were assayed in presence of PT-gliadin (1 µg/µL) through optical microscope evaluations at different time intervals (24–48–72 h post treatment, p.t.). As documented in Figure 3, at 24 h p.t, PT-gliadin spontaneously formed large extra-cellular aggregates. These aggregates were also visible in proximity or associated with plasma membranes and within large intra-cellular vesicles (upper right panel). On the contrary, the number and shape of vesicles were significantly reduced in untreated cells (left panels). At 48 h p.t., a certain degree of PT-gliadin induced toxicity was reported. Then, to evaluate the PT-gliadin extra- and intra-cellular traffic, GLIA-488 (1 µg/µL) was administered to growing Caco-2 cells. These cells were monitored through inverted fluorescent microscope evaluations (Figure 4). Soon after PT-gliadin incubation, i.e., from 30 min to 4 h, GLIA-488 was localized attached to plasma membranes and next, from 24 h p.t., GLIA-488 internalized into large vesicles. Then, following media renewal and after additional 24 h incubation, a certain degree of toxicity was shown and, as a consequence, damaged cells released fluorescent PT-gliadin aggregates into the extra-cellular environment. Of note, these de novo released GLIA-488 aggregates can be internalized into gliadin-untreated proliferating Caco-2 cells (data not shown).

**Figure 5:**
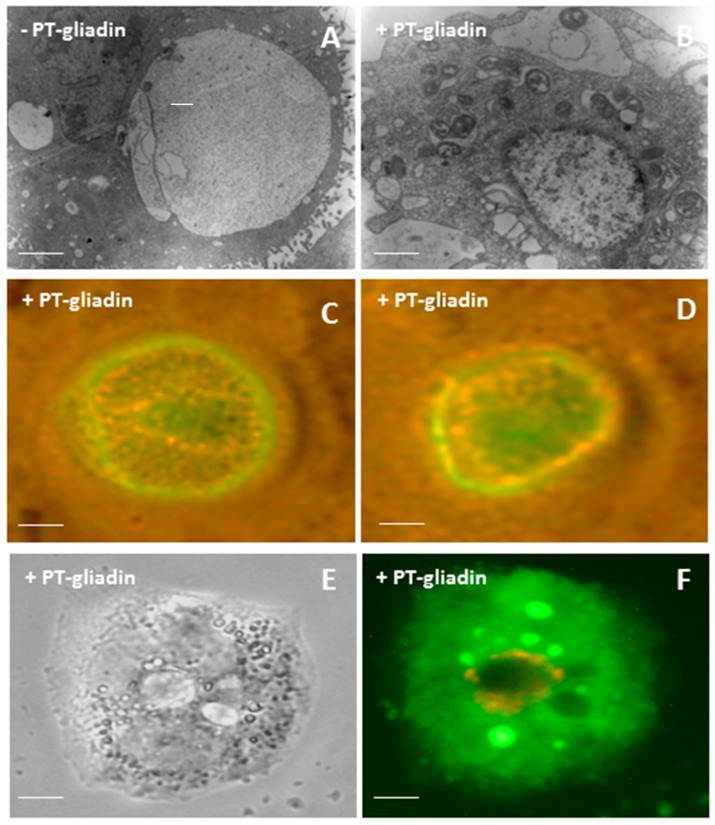
Ultrastructural and molecular characterization of intracellular large vesicles after PT-gliadin administration in Caco-2 cells. (**A**,**B**) TEM analysis of Caco-2 cells at 24 h after PT-gliadin administration (1 µg/µL) visualized using a Zeiss EM900 electron microscope. Scale bars = 2 µm. (**C**,**D**) Caco-2 cells incubated with PT-gliadin as above were transduced with the Premo Autophagy Sensor LC3B-GFP system, according to the guidelines and visualized using an inverted microscope Eclipse Nikon TS100, 100× oil immersion Plan Fluor objective. Scale bars = 2 µm (computer magnification); (**E**,**F**) For intra-vesicular pH content of Caco-2 vesicles, after PT-gliadin administration (1 µg/µL for 24 h), cells were incubated with acridine orange (1 µg/mL) for 10 min. Cells were then visualized under the optical microscope and using an inverted microscope Eclipse Nikon TS100, 40×, respectively. (scale bars = 10 µm).

To better characterize the observed large intra-cellular vesicles containing PT-gliadin aggregates, Transmission Electron Microscope (TEM) visualizations were performed. As reported (Figure 5A,B), Caco-2 cells stored PT-gliadin into large double membrane vesicles. These vesicles showed a marked expression of fluorescent LC3-II protein, 24 h after transduction with the Premo Autophagy Sensor LC3B-GFP system, containing a modified baculovirus vector expressing LC3-GFP [20] (Figure 5C,D). Then, acridine orange, a fluorescent dye that is permeable to the cell membranes and that accumulates in low-pH vesicles, was administered to growing Caco-2 cells previously incubated with PT-gliadin (1 µg/µL). As reported (Figure 5E,F), the intra-cellular vesicles, containing large amount of PT-gliadin aggregates displayed a relatively high pH content; differently, these vesicles were surrounded by red acidic vesicles, likely lysosomes. To further characterize the large intra-cellular vesicles showing PT-gliadin aggregates and to track the lysosome traffic and their distribution following PT-gliadin administration, Caco-2 cells were treated as above, and, after 24 h p.t. they were fixed and analysed for the autophagosome marker LC3 (Figure S1A) and for Lamp1 expression, a lysosomal integral membrane protein, by immunofluorescence (Figure S1B). Similarly to LC3, Lamp1 showed a marked accumulation at perinuclear foci that colocalized with gliadin protein signals. In addition, Lamp1 immunoblotting was performed and the subsequent normalization with BACT protein indicated a significant increase of the protein expression compared to PT-gliadin untreated Caco-2 cells (Figure S1C).

### 2.2. PT-Gliadin Affects Viability and Impairs Autophagy in Caco-2 Cells

**Figure 6:**
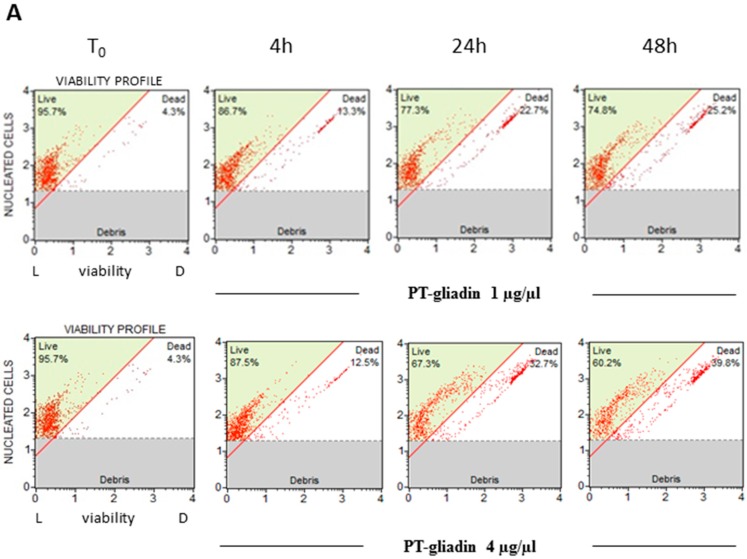

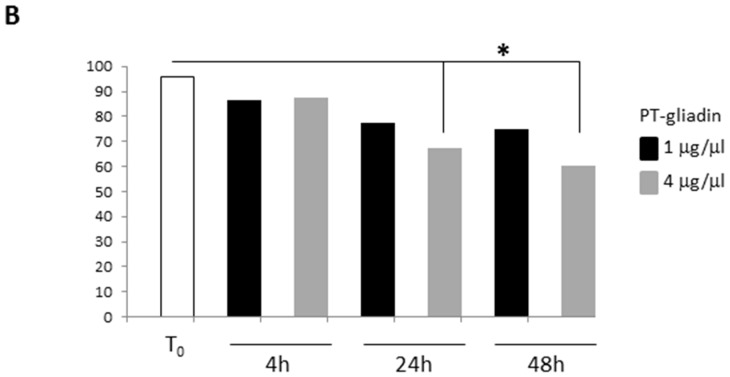
Viability assays after PT-gliadin administration in Caco-2 cells. (**A**) Cytofluorimetric plots of viability profiles (L: cells live; D: cells death) summarized in Panel (**B**) (asterisk indicates *p* < 0.05, Anova One-way, compared to T_0_ untreated sample).

**Figure 7:**
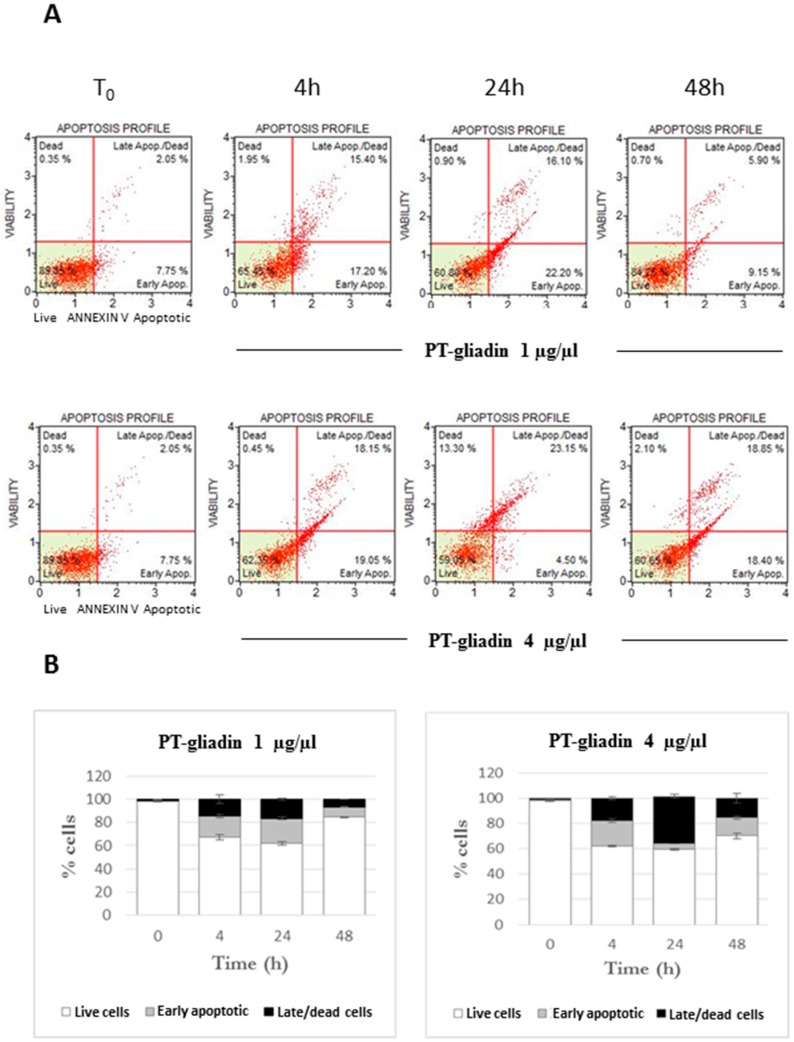
Apoptotic analysis of Caco-2 cells after PT-gliadin administration. (**A**) Cytofluorimetric plots of apoptotic profiles of Annexin V expression, summarized in Panel (**B**).

To investigate the effects of the peptic-tryptic digested gliadin on toxicity, Caco-2 proliferating cells were treated with PT-gliadin at different concentrations (1–4 µg/µL). Cell viability was measured at different times (T_0_–4–24–48 h p.t.) through the Cell Viability cytofluorimetric assay (Merck, Kenilworth, NJ, USA). The results pointed for time-dependent decreased trends of viability compared to untreated cells (T_0_), mostly at 48 h p.t. for both PT-gliadin concentrations employed (Figure 6). According to the viability data, Annexin V cytofluorimetric analysis of Caco-2 cells after PT-gliadin administration (using the above mentioned concentrations) highlighted an increase of early and late apoptotic induced cell death events from 4 to 48 h p.t. (Figure 7). PT-gliadin induced toxicity in Caco-2 cells was also documented by micronuclei evaluations. Cells were incubated or not- with PT-gliadin (1 µg/µL) for 24 and 48 h and next analysed at the different time intervals for the genesis of micronuclei. As reported (Figure S2), PT-gliadin significantly increased the formation of micronuclei at both time intervals investigated when compared to gliadin-untreated cells.

**Figure 8:**
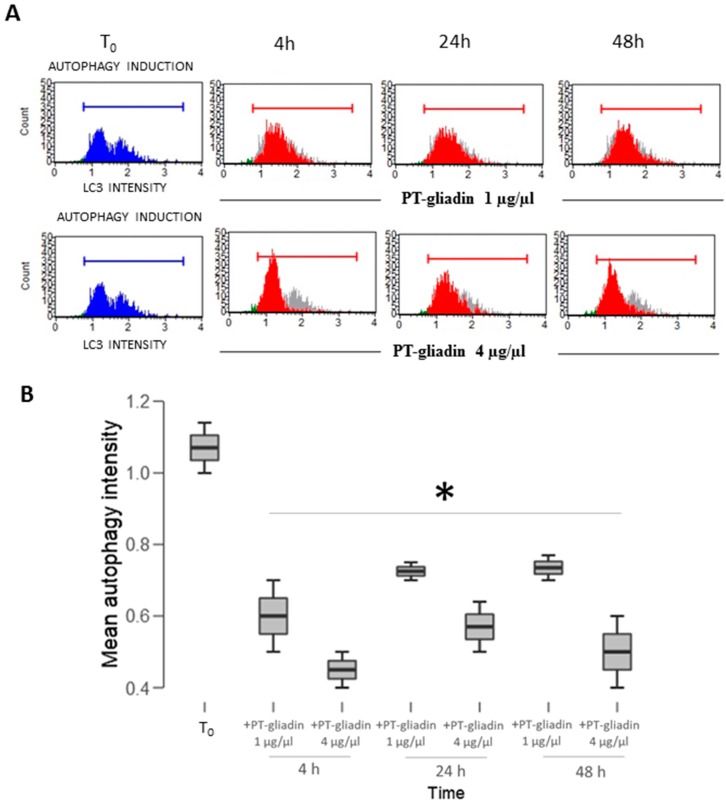
LC3-II expression in Caco-2 cells after PT-gliadin administration. (**A**) Cytofluorimetric plots representing LC3-II expression in Caco-2 cells incubated with 1 or 4 µg/µL of PT-gliadin at different time intervals (h); (**B**) Graphical summary of LC3-II expression trends. Asterisks indicate *p* < 0.05, Anova One-way, as compared to untreated NT samples at T_0_.

**Figure 9:**
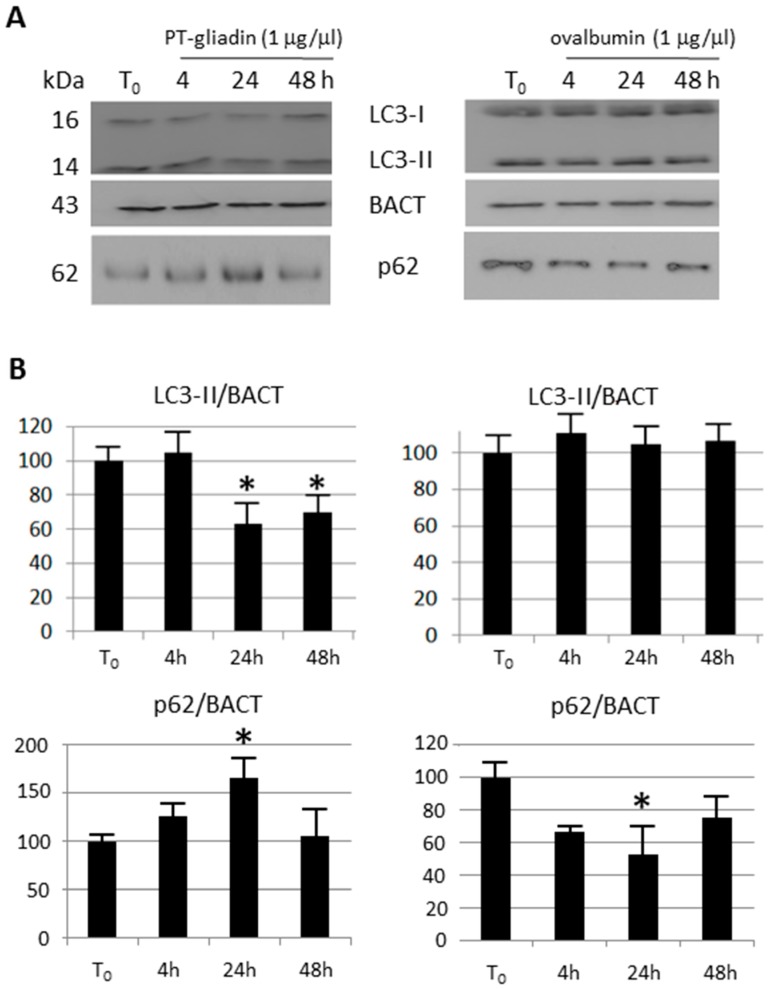
Immunoblotting analysis of LC3-II and p62 expression in Caco-2 cells after PT-gliadin or ovalbumine administration. (**A**) PT-gliadin (**left**) or ovalbumin (**right**, both at 1 µg/µL) were administered to Caco-2 cells. (**B**) LC3-II, p62 and BACT protein expression was analysed using immunoblotting and densitometric analyses. LC3-II and p62 were normalized to BACT levels as recommended [22]. Molecular weights (in kDa) are indicated. Asterisks indicate *p* < 0.05, Anova One-way, compared to T_0_ untreated sample.

To investigate the effect of PT-gliadin on the modulation of the autophagy process, Caco-2 cells were incubated with different amount of PT-gliadin and analysed at subsequent time intervals for LC3-II expression, a widely used autophagy-marker [21]. Specifically, Caco-2 cells were cultured in complete medium, with/without PT-gliadin (1–4 µg/µL); then, LC3-II expression was measured using the LC3B Autophagy cytofluorimetric assay (Merck) at different time intervals (4–24–48 h p.t.) (Figure 8A). As documented in the plots and summarized in Figure 8B, LC3-II protein displayed reduced expression trends at both concentrations as compared to untreated cells (T_0_). These results were confirmed by immunoblotting analysis after PT-gliadin administration (1 µg/µL); as a negative input control protein, identical amount of egg ovalbumin was administered. The expression of LC3-II was comparatively analysed in Figure 9, showing a time dependent decrease in PT-gliadin treated cells, when compared to untreated or ovalbumin-treated cells. On the other hand, p62 was partly cleared only after ovalbumin administration, while it accumulated following gliadin treatment.

### 2.3. the Effects of Autophagy Modulation in Caco-2 Cells Treated with PT-Gliadin

**Figure 10:**
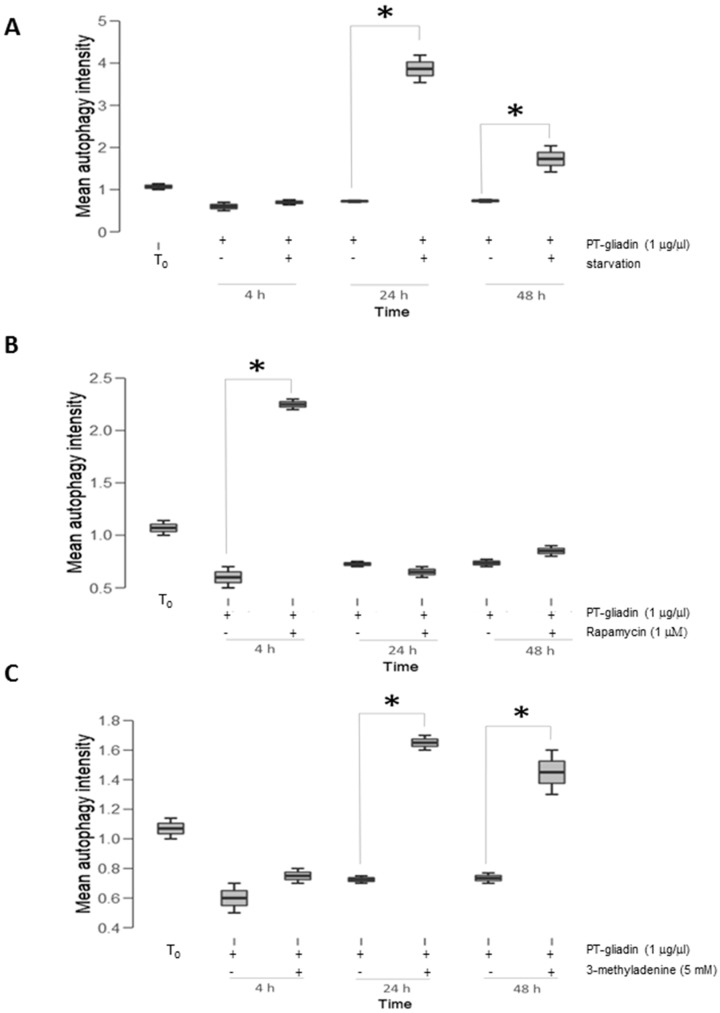
LC3-II expression following modulation of autophagy in Caco-2 cells incubated with PT-gliadin. LC3-II expression in Caco-2 cells treated with PT-gliadin (1 µg/µL), after starvation (**A**), rapamycyn (5 µM) (**B**) or 3-methyladenine (5 mM) (**C**) administration, during different time intervals (hours). Asterisks indicate statistical significance *p* < 0.05, Anova One-way, as compared to untreated NT samples at T_0_.

Then, Caco-2 cells were cultured as described, in HBSS medium to induce starvation [23] or treated with 5 µM rapamycin [24] or 5 mM 3-methyladenine (3-MA) [25] in the presence with PT-gliadin (1–4 µg/µL). LC3-II expression was measured as above by cytofluorimeter at different time intervals (4–24–48 h p.t.) (Figure 10). As reported, LC3-II expression displayed a marked increased expression trend for starvation and 3-MA treatments with statistical significant peaks at 24–48 h p.t.; differently, rapamycin produced a single peak of activation at four hours p.t. followed by a decreasing trend.

**Figure 11:**
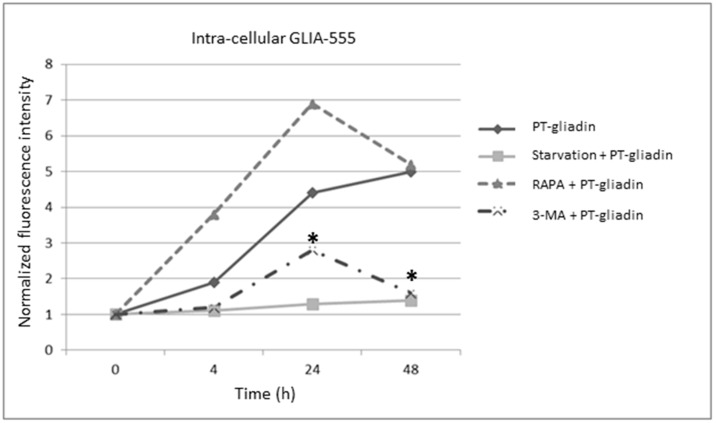
Intra-cellular fluorescent PT-gliadin (GLIA-555) content following autophagy modulation in Caco-2 cells. PT-gliadin was administered as fluorescent GLIA-555 (1 µg/µL) moieties and assayed at different p.t. intervals by cytofluorimetric evaluations, following different autophagy activation protocols (starvation using HBSS medium; rapamycin 5 µM; 3-MA 5 mM). Asterisks indicates statistical significance *p* < 0.05, Anova One-way, when compared to the respective time interval; of Caco-2 cells incubated with PT-gliadin.

To investigate the intra-cellular turnover of PT-gliadin content in Caco-2 proliferating cells, these were cultured in presence of fluorescent labelled PT-gliadin GLIA-555 (1 µg/µL) and subjected, as above, to starvation, rapamycin or 3-MA treatments. Thus, the intra-cellular fluorescence amount was quantified by cytofluorimetric analysis at different time intervals (4–24–48 h p.t.), with/without autophagy activation (Figure 11). As documented, the most effective treatments in reducing the intra-cellular GLIA-555 content were starvation induction and 3-MA administration; differently, rapamycin addiction produced a time-course increase of the intra-cellular amount of fluorescent PT-gliadin with a similar trend of PT-gliadin administration. 

**Figure 12:**
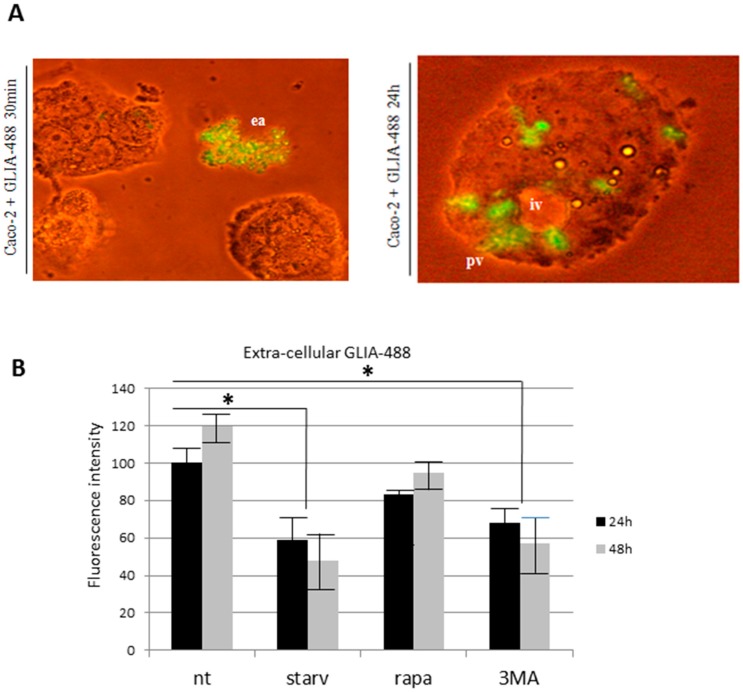
Extra- and intra-cellular evaluation of fluorescent PT-gliadin in Caco-2 cells. (**A**) Different time intervals of GLIA-488 administration (1 µg/µL) were reported (left, 30 min. p.t.: right, 24 h p.t.). Note a large autophagosome-like vesicle in the right Panel storing large fluorescent aggregates (ea: extra-cellular aggregates; pv: perimembrane exocytic vesicles; iv: intra-cellular vesicles). Scale bars = 10 µm; (**B**) GLIA-488 fluorescent labelled digested gliadin (1 µg/µL) was administered to growing Caco-2 cells in presence of starvation conditions, rapamycyn (5 µM) and 3-MA (5 mM) treatments. Media were collected and analysed by fluorimeter (ext. 492 nm–emis. 517 nm). Asterisks indicates statistical significance *p* < 0.05, Anova One-way, compared to untreated sample (nt). Fluorescence was reported as arbitrary units. SD bars (*n* = 3) are reported.

The quantitation of the extra-cellular amount of PT-gliadin was performed by GLIA-488 (1 µg/µL) administration to proliferating Caco-2 cells, followed by fluorimetric analysis of the collected media. Primarily, extra- and intra-cellular traffic of GLIA-488 was monitored using inverted fluorescent microscope in living Caco-2 cells. As reported in Figure 12, 30 min after GLIA-488 administration, large extra-cellular fluorescent aggregates were visible (Panel, A, left), while at 24 h p.t., GLIA-488 signals were visible in proximity of large intra-cellular vesicles or in correspondence to peri-membrane exo/endocytic vesicles (Panel A, right). Then, a fluorometric analysis of the extra-cellular content of GLIA-488 during different time intervals (24–48 h p.t.) was performed in normal growing vs. autophagy-modulated Caco-2 cells (Figure 12B). As reported, the most effective and statistically significant GLIA-488 extra-cellular content reductions in the media were scored during the starvation-induced scheme, and following 3-MA treatment, when compared to untreated (NT) sample. Differently, rapamycin treatment produced only a slight reduction of the release of GLIA-488 in the extra-cellular medium.

**Figure 13:**
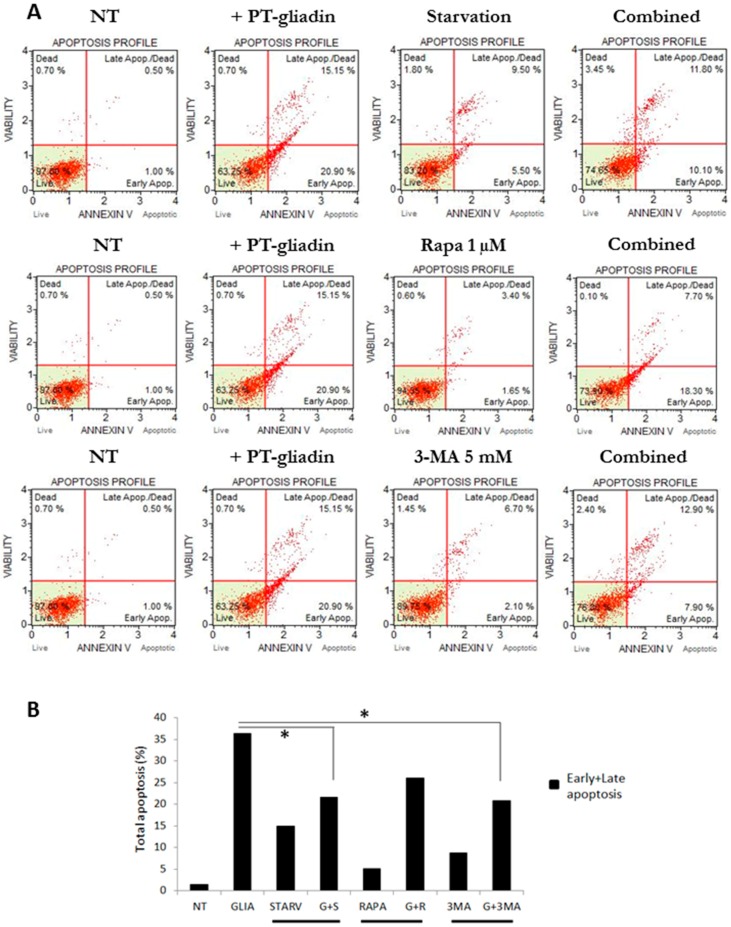
Apoptotic analysis of Caco-2 cells after PT-gliadin administration and autophagy modulation protocols. (**A**) Cytofluorimetric plots of apoptotic profiles of Annexin V expression, for single and combined treatments (starvation, rapamycin and 3-methyladenine) summarized, as total apoptotic events (Early + Late) in Panel (**B**).

Then, to discern the role of autophagy following PT-gliadin administration, one of the key executor autophagy-related gene, i.e., *BECN1*, was silenced using a pool of validated siRNA moieties in Caco-2 cells, challenged with fluorescent PT-gliadin (GLIA-488): firstly, the effect of the transfection of si*BECN1* on protein expression was monitored at 48 and 72 h p.t. (Figure S3A). Then, as previously described, the cells were incubated with GLIA-488 (1 µg/µL) fluorescent proteins and were transfected with si*BECN1* molecules. Extra-cellular media at 24 and 48 h p.t. were collected and analysed by fluorimetric analysis (Figure S3B). As reported, *BECN1* silencing did not alter the extra-cellular fluorescent amount of PT-gliadin at both of the time intervals investigated. Next, the assayed autophagy modulatory protocols (i.e., starvation, rapamycin, and 3-MA) were compared in their capability to reduce PT-gliadin induced toxicity through the analysis of the apoptotic profile by Annexin V cytofluorimetric analysis. As reported (Figure 13), starvation induction and 3-MA (5 mM) administration exhibited, in concomitant with PT-gliadin (1 µg/µL) incubation, significant effects on the reduction of the total apoptosis events as compared to the PT-gliadin treatment.

## 3. Discussion

In this work, in vitro experiments were conducted to elucidate the autophagy involvement in the cellular response to PT-gliadin administration. The working hypothesis was that the autophagy process, in its catabolic function of unselective degradation of bulk exogenous proteins, might reduce the toxicity of gliadin proteins. To this purpose, after the evaluation of the effect of PT-gliadin on the endogenous autophagy status of the cells, different autophagy-modulators were assayed to induce a favorable PT-gliadin degradation.

As a first approach, Caco-2 cells, which partly mimic polarised epithelial transport showing physiological similarities with small bowel enterocytes [26,27], were treated with enzymatically digested peptic-tryptic gliadin (PT-gliadin) peptides at two different concentrations and cells were monitored at different interval times through optical microscope observations. A marked, time-dependent, toxicity was observed and the reduction of cell viability was confirmed by cytofluorimetric viability and apoptotic assays and through the analysis of micronuclei induction.

Optical, fluorescent, and TEM microscope observations showed that PT-gliadin can form sticky and large extra-cellular aggregates, as already reported [28]. This biochemical property can be explained by the presence of glutamine and proline-rich repetitive regions in the N-terminal of gliadin protein. The importance of these repetitive regions in determining the sticky properties of gliadin was demonstrated by the use of deletion mutant of γ-gliadin. These mutants, lacking the entire N-terminal region, are not able to aggregate [29]. As already demonstrated, Caco-2 cells are able to endocyte gliadin peptides and segregate them into early endosomal compartments [22]. Concordantly, our results using fluorescent labeled PT-gliadin tracked the internalization, the storage into large vesicles, and, finally, the extra-cellular release of these proteins. To initially characterize these large intra-cellular vesicles, different microscope analysis were performed. Firstly, ultrastructural TEM analysis highlighted the storage of PT-gliadin into large double membrane vesicles. Then, the cells were incubated with the acidotrophic acridine orange dye after PT-gliadin uptake. This dye is useful to visualize acidic vesicles, i.e., lysosomes and autophagosomes, because of the emission changes dependent to pH conditions. In particular, it is known that acridine orange dye emits orange/red light in presence of the low pH inside these vesicles, lysosomes, or autophagosomes, according to their shape and size [30]. Our results documented thick and rough particles within the lumen of large vesicles, positive for the autophagosome marker LC3-II; LC3-II is the only known protein to form a stable association with the membrane of autophagosomes. It is known to exist in two forms: LC3-I, which is found in the cytoplasm and LC3-II, which is membrane-bound and is converted from LC3-I, to initiate the formation and lengthening of the autophagosome [21].

Our data showed also an increase of the recruitment of lysosomes, the marked expression of the baculovirus-derived LC3B-GFP or LC3-II protein in correspondence of vesicular membranes and the relatively high intra-vesicular pH values suggested that these vesicles were defective late autophagosomes in their capability to digest PT-gliadin. This defect in protein degradation was also highlighted by the p62 expression profile. Furthermore, the analysis of the autophagy flux by Bafilomycin A1 treatment (10 nM, 6 h before each LC3-II evaluation), as recommended by current autophagy guidelines [21], did not show significant increase in LC3-II expression in PT-gliadin-treated cells as compared to PT-gliadin-untreated controls (data not shown). Of note, vesicles containing PT-gliadin aggregates were detected days after the incorporation, suggesting that Caco-2 were not able to complete the degradation of excess amounts of PT-gliadin (data not shown). 

Autophagy is conventionally described as a catabolic pathway where the cytoplasmic material sequestered by autophagosomes to be degraded [31]. As already stated, this process plays a crucial role in several physiological and pathological mechanisms, such as protein aggregates degradation [32,33]. In our case, once internalized, PT-gliadin biochemical behavior resembles that of the toxic proteins causing protein conformational diseases. Our results provided evidence that PT-gliadin was endocyted by cells and addressed to autophagosomes for its degradation, but, despite the presence of these specific vesicles, cells were not able to completely metabolize and degrade these peptides. On the other side, it is known that impairment of autophagy leads to exocytosis of peptides that cannot be degraded, as in the case of Parkinson disease, through a secretory pathway named “exophagy”, which involves the intermediate compartments of autophagy [34]. Moreover, the secreted peptides could be internalized by other cells thus perpetuating the toxic effect of peptides to neighboring cells [35].

To investigate the effect of PT-gliadin on the modulation of the autophagy process, Caco-2 cells were incubated with different amount of PT-gliadin and analysed at subsequent time intervals for LC3-II expression, a well-documented autophagy-marker [21]; the behaviour of the cells were also compared with egg-ovalbumin, an exogenous protein normally used as a negative control in endocytic and toxicity assays [36]. Differently to ovalbumin treatment, LC3-II displayed down-regulated expression profiles at both PT-gliadin concentrations employed. Thus, autophagy modulation experiments have been carried out using well established approaches, specifically the induction of starvation and the administration of rapamycin or 3-methyladenine. Starvation is a well-documented environmental condition that promotes autophagy responses in different cells [37,38,39]. Conversely, rapamycin is a widely used compound able to induce autophagy in a large variety of cells through an inhibitory effect on the authophagy negative regulator mTOR complex [40]. Differently, 3-methyladenine has been widely used as autophagy inhibitor based on its effect on class III PI3K activity, which is known to be essential for induction of autophagy. However, Wu and collaborators [25] reported that 3-methyladenine promoted an increase of the autophagy flux when cells are treated under nutrient-rich conditions with a prolonged period of treatment. Autophagy induction was preliminary confirmed for the scheduled treatments evaluating the expression levels of LC3-II protein by cytofluorimetric analysis in Caco-2 cells after PT-gliadin administration. Specifically, the most effective and lasting treatments in rescuing LC3-II expression were starvation and 3-methyladenine (5 mM). Differently, rapamycin (5 µM) administration induced a relatively short interval time of LC3-II up-regulation, while, at higher doses (i.e., 10 to 20 µM), a certain degree of cytotoxicity was scored. Similarly, other pharmacological schemes, either vaccination, chronic, or acute, of administration of the two drugs (i.e., rapamycin or 3-methyladenine) produced lesser effects in LC3-II induction or induced proliferation cellular arrest (data not shown). Then, to evaluate the effect of autophagy modulation schemes on the specific target of the PT-gliadin molecules, we specifically developed quantitative approaches to measure differences in the intra- or extra-cellular amount of PT-gliadin. The former measurement was based on the cytofluorimetric analysis of a PT-gliadin molecules conjugated with the Alexa Fluor 555 dye, while the latter on the fluorimetric amount of PT-gliadin Alexa Fluor 488 complex in the extra-cellular media. Accordingly to LC3-II expression profiles, the most effective treatments in both reducing intra- and extra-cellular fluorescent PT-gliadin amount were the induction of starvation and 3-methyladenine administration. Importantly, when these treatments were combined with PT-gliadin administration in Caco-2 cells, they showed reduced levels of cytotoxicity, as documented by apoptotic assays.

Altogether, in this contribution, we have highlighted the involvement of the crucial autophagy process in cells challenged with enzymatically digested gliadin peptides. To date, only few contributions suggested a functional link between autophagy and celiac disease (CD). In particular, Rajaguru and collaborators [41] showed histological improvement in duodenal biopsies associated with reduction in activated dendritic cells expressing autophagic markers, suggesting a possible role in the pathogenesis of autoimmune disorders, like CD. More recently, we have reported a down-regulation of the important autophagy-executor gene *BECN1*, accomplished by the increase of one of its negative microRNA regulators (i.e., miR30a) in a cohort of pediatric CD patients as compared to age-related controls [42]. On the other side, the possible role of defective autophagic process has been proposed in the development of Crohn`s disease, which is an inflammatory bowel disease that may affect the gastrointestinal tract [43]; furthermore, the key role for the autophagy gene *Atg16l1* in mouse and human intestinal Paneth cells was previously documented [44]. Among our results, we reported that autophagy induction through starvation gave a proliferative advantage to cells increasing PT-gliadin degradation, reduced its exocytosis, thus limiting the toxic effects of these peptides on neighboring cells. One well-recognized way of inducing autophagy is by food restriction, which up-regulates autophagy in many organs including the brain [45]. Recently, the health benefits of fasting-based diets have been highlighted against oxidative stress and inflammation. In particular, it was also demonstrated that intermittent or periodic fasting in humans helps to reduce obesity, hypertension, rheumatoid arthritis, and confers protection to cancer [46]. Moreover, it was described that fasting and particular protein dietary regimens optimize longevity, sensitize tumors to chemotherapy in mice models and even in humans [47].

Next experiments are directed to better characterize the autophagic status of differentiated Caco-2 cells, reducing their morphological and genetic heterogeneity, and constituted by monolayers of polarized cells, coupled by tight junctions and expressing several morphological and functional features of small intestinal enterocytes.

In conclusion, these considerations, along with our preliminary results, might add the autophagy process, referred to as cellular “cleansing” natural system, in the growing list of molecular actors that play critical roles in the pathogenesis of CD and therefore giving rise to a novel therapeutic strategies. Very recently, “drug-reposition” strategies (reviewed in [48]), i.e., the concept that “conventional” agents used to treat diseases other than cancer can have therapeutic effects by activating/suppressing autophagy, has attracted increasing attention because the safety profiles of these medicines are well known. Antimalarial agents, such as artemisinin and disease-modifying antirheumatic drug, are novel examples of drug re-positioning that affect the autophagy regulation for wide therapeutic use.

## 4. Materials and Methods

### 4.1. Cell Culture and Autophagy Modulation Protocols

Caco-2 cells from American Type Culture Collection (ATCC) were cultured and maintained in DMEM medium (Euroclone, Milano, Italy), supplemented with (or without in starvation experiments) 10% FBS, 100 units/mL penicillin, 0.1 mg/mL streptomycin, and 1% l-glutamine, and kept at 37 °C in a 5% CO_2_/95% air atmosphere. Most of the experiments were performed in 6–12–24 multi-well plates with different cell concentrations, depending on each experiment requirements. For starvation induction, Caco-2 cells were grown in complete medium with digested gliadin, then, after 24 h, were washed twice with PBS and incubated with HBSS medium (Euroclone) for different time intervals. As before, cells incubated with digested gliadin for 24 h in complete medium, were then treated with rapamycin (5 µM) or 3-methyladenine (Sigma, St. Louis, MO, USA) (5 mM) and monitored at different interval times. 

### 4.2. Peptic and Trypsin Digested Gliadin (PT-Gliadin) Preparation And Fluorescent Labeling

Gliadin from wheat (Sigma) was digested, as described [18]. In detail, whole gliadin (1 g/mL) was firstly dissolved in 500 mL 0.2 N HCl for two hours at 37 °C with 1 g pepsin (Sigma). The resultant peptic digest was further digested by addition of 1 g trypsin (Sigma), after pH adjusted to 7.4 using 2 N NaOH; next, the solution was incubated at 37 °C for four hours with a vigorous agitation. Finally, the mixture was boiled to inactivate enzymes for 30 min and was stored at −20 °C (hereafter referred as PT-gliadin). Ovalbumin (Sigma) at 1 g/mL was used as an additional external protein for internalization and cell toxicity studies. PT-gliadin or ovalbumin were then administered directly to the cells. For PT-gliadin Alexa Fluor 488 or 555 labeling, purification of unlabeled dyes was performed using affinity chromatography-purification G50 columns (GE Healthcare, Little Chalfont, UK). The resulted proteins were resuspended in PBS and quantified through Qubit Fluorometer (Invitrogen, Carlsbad, CA, USA). Then, PT-gliadin or ovalbumin (100 µg each) proteins were labeled with Alexa Fluor 488 or Alexa Fluor 555 by specific Alexa Fluor Microscale labelling kits (ThermoFisher, Waltham, MA, USA), according to manufacturer’s instructions.

### 4.3. Transmission Electron Microscopy (TEM) Analysis of PT-Gliadin Aggregates in Caco-2 Cells

PT-gliadin aggregates were visualized by TEM as described [42]. In detail, 20 microliter drops of PT-gliadin (1 µg/µL) in PBS on a Parafilm (Sigma) sheet, and a 300-mesh nickel grid (covered with a Formvar-carbon film) were floated onto the drops for five minutes. The grids were then blotted with filter paper and negatively stained with a 2% (*v*/*v*) phosphotungstic acid solution, pH 7.0, for 60 s and observed on a Zeiss EM900 electron microscope (Zeiss, Oberkochen, Germany) operating at 80 kV.

For Caco-2 ultrastructural analysis, TEM was performed according to [49]. After 24 h in presence with PT-gliadin (1 µg/µL), about 10^6^ cells were washed twice with PBS, harvested by centrifugation at 800 rpm for 3 min and fixed with 2% (*v*/*v*) glutaraldehyde in DMEM, for two hours at room temperature. Cells where then rinsed overnight in PBS (pH 7.2) and then post-fixed in 1% (*w*/*v*) aqueous OsO_4_ for two hours at room temperature. Cells were pre-embedded in 2% agarose in water, dehydrated in acetone, and finally embedded in epoxy resin (Electron Microscopy Sciences, EM-bed812). Ultrathin sections (50–60 nm) were collected on formvar-carbon-coated nickel grids and stained with uranyl acetate and lead citrate. The specimens were observed with a Zeiss EM900 transmission electron microscope (TEM) equipped with a 30 µm objective aperture and operating at 80 kV.

### 4.4. Immunofluorescence Analysis

Immunofluorescence assays were performed as reported [50]. Specifically, primary anti-gliadin (Abcam, Cambridge, UK) and anti-Lamp1 (Santa Cruz Biotechnology, Dallas, TX, USA) antibodies were diluted 1:30 in T-PBS 5% (*v*/*v*) and incubated for one hour at room temperature. Species-specific Alexa Fluor 488 and Alexa Fluor 633 (Molecular Probes, Eugene, OR, USA) secondary conjugated antibodies (1:100 in T-PBS 5%) were then incubated for an additional one hour at room temperature. Cells grown on coverslips were washed three times with PBS and fixed with 4% (*v*/*v*) paraformaldehyde-PBS, pH 7.4, for 15 min at room temperature. Coverlips were then washed three times with PBS and were treated with ProLong Gold antifade reagent with DAPI (Invitrogen), according to the manufacturer’s instructions and finally mounted onto microscope glass slides. Fluorescence signals were detected using a fluorescence light inverted microscope (Nikon Eclipse TS100, Tokio, Japan) with a Plan Fluor 100× oil immersion objective. 

### 4.5. Acridine Orange Staining

Monitoring and visualization of acidic vesicles was performed using an acidotropic dye, i.e., acridine orange (ext. 500 nm–emis. 525 nm). Acridine orange was added at the final concentration of 1 µg/mL in Caco-2 cells in presence or absence of PT-gliadin (1 µg/µL), 10 min before its fluorescent visualization. Red fluorescent spots are indicative of a higher intravesicular acidic content. Autophagosomes and lysosomes can be distinguished according to their shape and size since the former are more heterogeneous and bigger in dimensions [30]. Fluorescence signals were detected using a fluorescence light inverted microscope (Nikon Eclipse TS100) with a Plan Fluor 100× oil immersion objective. 

### 4.6. Lc3b-Gfp Autophagosome Analysis

For autophagosome detection, Caco-2 cells were seeded at the density of 5 × 10^4^ cells/well in 24-multiwell plates and were incubated with PT-gliadin (1 µg/µL). After 24 h, cells were transducted with BacMam LC3B-GFP with a multiplicity of infection (MOI) equal to 30, according to Premo Autophagy Sensor kit (ThermoFisher). LC3B-GFP signals were monitored at 24 and 48 h p.t. using and inverted fluorescence microscope (100× magnification, Plan Fluor oil immersion objective Eclipse Nikon TS100), as described [20].

### 4.7. Immunoblotting Analysis

Caco-2 cells, 5 × 10^4^ cells/well, were cultured in 24-multiwell plates in the presence/absence of PT-gliadin, ovalbumin (both at 1 µg/µL) and collected at different interval times. Cells were trypsinized, centrifuged 10 min at 14,800 rpm, and dried pellets were kept at −80 °C upon protein extraction. Cells were lysated in ice-cold RIPA buffer (150 mM NaCl, 50 mM Tris-HCl pH 8.0, 0.5% sodium deoxycholate, 1% Nonidet P40, 0.1% sodium dodecylsulphate) and supplemented with Complete Mini protease inhibitor cocktail 7X (Roche, Basel, Switzerland). Protein extracts were quantified using the Quant-it Protein Assay Kit (Invitrogen). Proteins were added with LaemmLi sample buffer (2% SDS, 6% glycerol, 150 mM β-mercaptoethanol, 0.02% bromophenol blue and 62.5 mM TRIS-HCl pH 6.8), denatured for five minutes at 95 °C and separated on 15% SDS-PAGE according to the protein size. After electrophoresis, proteins were transferred onto nitro-cellulose membrane Hybond-C Extra (GE Healthcare), using the semi-dry blotter TE70 PWR apparatus (GE Healthcare). Membranes were blocked 1 h with 5% (*w*/*v*) non-fat milk in TBS (138 mM NaCl, 20 mM Tris-OH pH 7.6) containing 0.1% Tween 20 and incubated over-night at 4 °C with primary antibodies LC3-II and BECN1 (Cell Signaling, Danvers, MA, USA) diluted at 1/3000 in 5% non-fat milk in TBS, whereas BACT (Cell Signaling) was diluted at 1/10,000 in 5% non-fat milk in TBS. Species-specific peroxidase-labeled ECL secondary antibodies (Cell Signaling, 1/10,000 dilution) were used in 5% non-fat milk in TBS, as described [51]. Protein signals were revealed using the ECL Prime Western Blotting Detection Kit (GE Healthcare) by means of Chemidoc MP (Biorad, Hercules, CA, USA) instrument. Protein expression was quantified by densitometric analysis with ImageJ software [52].

### 4.8. Fluorimetric and Cytofluorimetric Analysis of Extra- And Intra-Cellular PT-Gliadin Content

For extra-cellular PT-gliadin assays, the media of Caco-2 cells, previously incubated with Alexa-Fluor 488 labeled PT-gliadin, were collected and quantified by Qubit Fluorometer (Invitrogen, ext. 492 nm–emis. 517 nm) at different times after fluorescent PT-gliadin administration and following the in vitro modulation schemes of the autophagy process. In detail, cells were seeded at the density of 2 × 10^4^ cells/well in 24-multiwell plates and were incubated for 24 h with Alexa-Fluor 488 labeled PT-gliadin (1 µg/µL). Cells were then washed three times with PBS and the media were replaced with complete DMEM FBS 10%. After additional 24 and 48 h, media were again collected and assayed in triplicates for fluorescent content. 

Similarly, for intra-cellular fluorescent PT-gliadin evaluations, Caco-2 cells (2 × 10^4^ cells/well) in 24-multiwell plates were incubated for 24 h with Alexa Fluor 555 labeled PT-gliadin (1 µg/µL). Cells were washed three times with PBS, trypsinized, and cytometrically assayed by Muse Cell Analyzer (Merck) in triplicates.

### 4.9. Apoptosis and Autophagy Cytofluorimetric Analysis after PT-Gliadin Administration

Live, dead, early-, or late-apoptotic Caco-2 cells were quantified using the Muse Annexin V & Dead Cell Assay (Merck) as specified. Briefly, cells (2 × 10^4^ cells/well) in 24-multiwell plates were incubated for 24 h with Alexa-Fluor 555 labeled PT-gliadin (1 µg/µL). Then, cells were washed three times with PBS, trypsinized, resuspended in 1% BSA (*v*/*v*), and 1 volume of Annexin V reagent was added and finally incubated at room temperature for 20 min and next analysed in triplicates using the Muse Cell Analyzer (Merck); for autophagy detection, the Muse Autophagy LC3-antibody based kit (Merck) was adopted: in this case, after fluorescent PT-gliadin incubation, cells were permeabilized and incubated on ice for 30 min with the anti-LC3 mouse monoclonal Alexa Fluor 555 conjugated antibody, according to the manufacturer’s protocol. Then, the intracellular LC3 fluorescence was assayed in triplicates using the Muse Cell Analyzer (Merck).

### 4.10. Micronuclei Assay

About 3 × 10^4^ Caco-2 cells were grown onto glass slices for 24 h in the presence/absence of PT-gliadin (1 µg/µL). Cells were then fixed at 24 and 48 h p.t. as follows: two washes with PBS, 15 min in KCl 75 mM, three washes of 15 min each in ice-cold methylacetic acid. Glasses were then mounted with DAPI and 1000 nuclei per sample were counted using and inverted fluorescence microscope (100× magnification, Plan Fluor oil immersion objective Eclipse Nikon TS100), according to guidelines [53].

### 4.11. BECN1 Sirna Assay and Transfection

To silence *BECN1* expression, Caco-2 cells, 2 × 10^4^ cells/well in 24-multiwell plates, were transiently transfected with 0.5 µL/well Lipofectamine 3000 reagent (Invitrogen) reagent coupled with a pool of validated si*BECN1* (si16537, si16538 and si16539, Ambion) sequences at the final concentration of 2.5 µM each. After 24 h post transfection, PT-gliadin (1 µg/µL) was added to the transfected and not transfected cells and BECN1 protein expression was monitored by immunoblotting at 48 and 72 h p.t. 

## Supplementary Materials

Supplementary materials can be found at www.mdpi.com/1422-0067/19/2/635/s1.
