# Supplementary material for: The In Vitro Effects of Enzymatic Digested Gliadin on the Functionality of the Autophagy Process
Source: Int J Mol Sci. 2018 Feb 23;19(2):635. doi: 10.3390/ijms19020635 (PMC5855857; doi:10.3390/ijms19020635)
Supplement: Supplementary file 1 [file ijms-19-00635-s001.pdf]

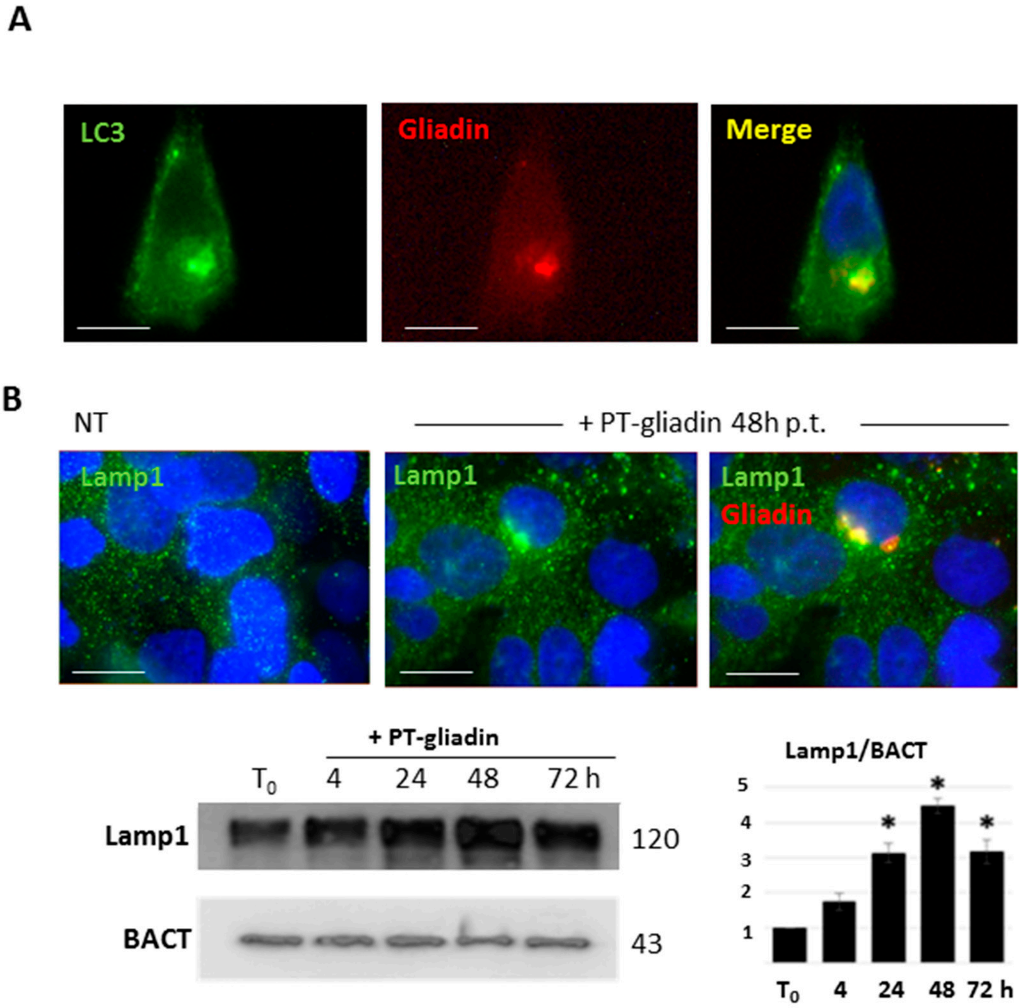


**Supplementary Figure 1** LC3 and Lamp1 expression in Caco-2 cells after PT-gliadin administration. (**A,B**) Immunofluorescence analysis of LC3 or Lamp1 (green) and gliadin (red) expression, visualized using an inverted microscope Eclipse Nikon TS100, 100X oil immersion Plan Flor objective. Scale bars=10 μm. (**C**) Immunoblotting expression and densitometric analysis of Lamp1 normalized with BACT housekeeping values. Asterisks indicate p<0.05, Anova One-way, compared to $T_0$ untreated sample.

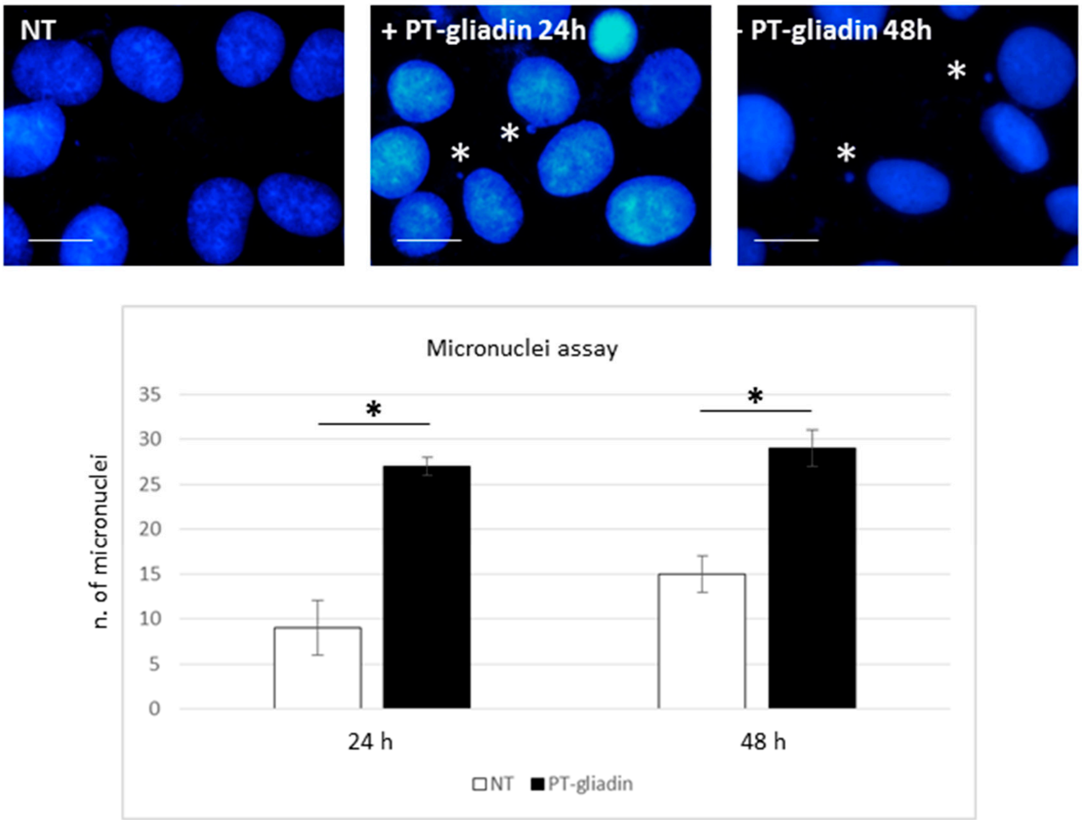


**Supplementary Figure 2.** Micronuclei formation after PT-gliadin administration in Caco-2 cells. Fluorescent DAPI staining and analysis of the number of micronuclei in Caco-2 cells treated with PT-gliadin (1 µg/µl). For each condition, 1000 nuclei were considered. Scale bars=10 µm. Asterisks indicates $p<0.05$, Anova One-way, compared to NT untreated samples.

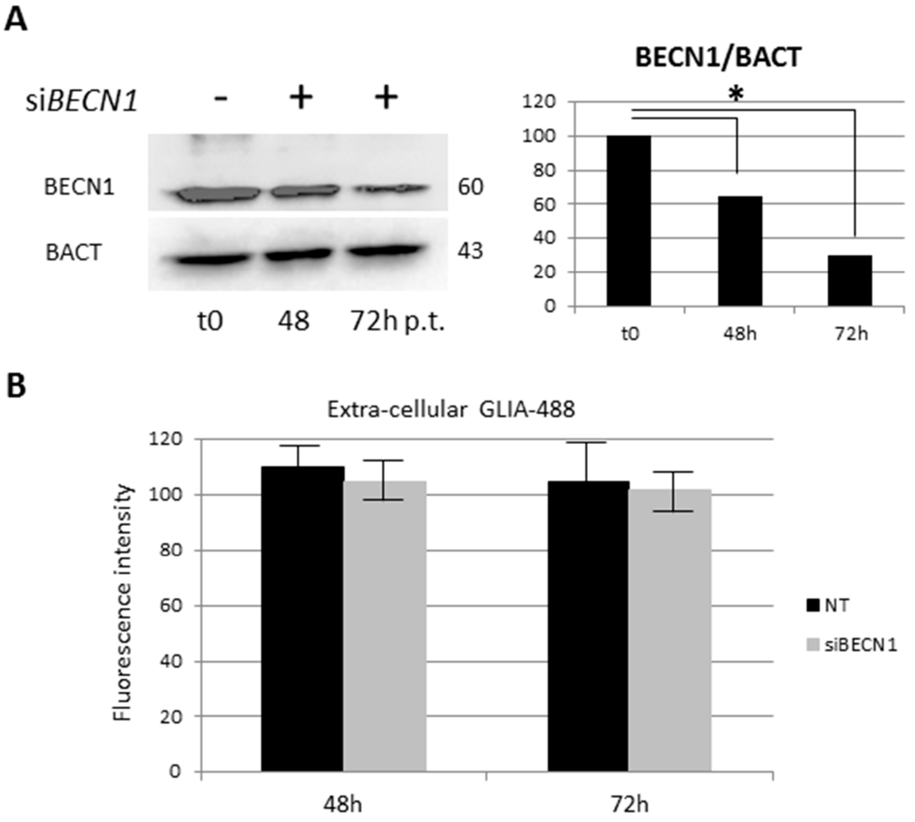


**Supplementary Figure 3.** Effect of *BECN1* silencing on Caco-2 cells after PT-gliadin administration. PT-gliadin (1 μg/μl) was administered to Caco-2 cells, transfected with a pool of validated si*BECN1* molecules. (**A**) Immunoblotting and densitometric analysis of BECN1 protein expression. (**B**) Collected media were analysed by fluorimeter (ext. 492 nm – emis. 517 nm). Asterisk indicates statistical significance $p<0.05$, Anova One-way, compared to untreated sample (nt). Fluorescence was reported as arbitrary units. SD bars (n=3) are reported.
